# Microplastic separation and enrichment in microchannels under derivative electric field gradient by bipolar electrode reactions

**DOI:** 10.1038/s41598-024-54921-0

**Published:** 2024-02-26

**Authors:** Zhenrong Sun, Chicheng Ma, Chengjiao Yu, Zirui Li

**Affiliations:** School of Mechanical Engineering, Hebei University of Technology, Tianjin, 300401 China

**Keywords:** Microplastic particles, Bipolar electrodes, Microfluidics, Finite element simulation, Chemical engineering, Pollution remediation

## Abstract

The decomposed plastic products in the natural environment evolve into tiny plastic particles with characteristics such as small size, lightweight, and difficulty in removal, resulting in a significant pollution issue in aquatic environments. Significant progress has been made in microplastic separation technology benefiting from microfluidic chips in recent years. Based on the mechanisms of microfluidic control technology, this study investigates the enrichment and separation mechanisms of polystyrene particles in an unbuffered solution. The Faraday reaction caused by the bipolar electrodes changes the electric field gradient and improves the separation efficiency. We also propose  an evaluation scheme to measure the separation efficiency. Finite element simulations are conducted to parametrically analyze the influence of applied voltages, channel geometry, and size of electrodes on plastic particle separation. The numerical cases indicate that the electrode-installed microfluidic channels separate microplastic particles effectively and precisely. The electrodes play an important role in local electric field distribution and trigger violent chemical reactions. By optimizing the microchannel structure, applied voltages, and separation channel angle, an optimal solution for separating microplastic particles can be found. This study could supply some references to control microplastic pollution in the future.

## Introduction

Plastic products have been widely utilized in people’s daily lives because of their properties of lightweight and corrosion resistance, However, plastic products are difficult to degrade in the natural environment for their stable chemical properties, leading to their long-term persistence in the environment. Microplastics are some plastic microbeads with smaller sizes than 5 mm compared to sizes of plastic, which are easier to residue in the environment. As time goes on, microplastics cumulatively and directly damage soils and crops due to residue in the soil^1^. Additionally, microplastics cause organism damage, trigger biological behaviors and form compound pollution in marine ecosystems^2^. The abundance of microplastic particles can be as high as 10,200 particles per cubic meter in certain nearshore and estuarine areas of China^3,4^. More recent evidence shows the expanding worldwide manufacturing and usage of microplastic has harmed human health^5–7^. Therefore, the control of microplastic pollution plays a vital role in the environment and humans. It is crucial for coordinating pollution reduction and carbon neutrality efforts.

There are many different types of microplastic particles, which have different properties. Researchers mainly considered three important characteristics: density, buoyancy, and size because these  properties serve as a reference for the selection of different separation methods. For example, polyethylene and polypropylene particles, whose density are less than the water, often use density separation methods. Scientists used extraction methods to separate medium-sized microbeads such as 20–25 μm, but it is different to deal with small-size microbeads^8^. Although various approaches have been put forward to remove microplastic particles from the environment, especially from the water environment^9–11^, the study gap of characteristics of microbeads restricted the development of these approaches. In addition, limited application scenarios and high costs are also the reasons that limit development. Therefore, there is a need to explore novel methods for particle treatment.

The surface functional groups of microplastic particles are altered by environmental factors, so the electronegativity of particles is enhanced^12^. Because of the wide size range and inherent electronegativity of microplastic particles, microfluidic technology holds a unique advantage in the enrichment, separation, and classification of charged particles. Microfluidic technology is a promising approach and it is worth exploring in dealing with microplastics. Microfluidic technology has many applications in the fields of biology and chemistry. As microfluidic technology has evolved, microfluidic separation technology is divided into passive separation technology and active separation technology. Passive microfluidic separation technology is a method that utilizes inertial lift and secondary flow resistance to achieve particle separation. Many researchers have focused on the structure design methods of inertial microchannels, including the addition of obstacles, variable cross-sectional width, and spiral channel design^13,14^. Previous work has led to the successful design of many inertial microchannels and has made significant progress in the advancement of inertial microchannels, but has failed to explore the underlying mechanisms of inertial microchannel structures^15^. Existing structural designs mostly relied on empirical knowledge and lacked theoretically guided design steps. This complexity in the design process and specificity of most inertial microchannels limited the application scenarios.

In contrast to passive microfluidics which relies on structures to process particles, active microfluidic separation technology controls the flow of cells or particles by adding external fields such as acoustic, thermal, magnetic, or electric fields in the microchannel^16^. Based on their well-established theoretical basis and the ability to effectively alter fluid velocity, active microfluidic separation technology controls particles or cells greatly^17^. Several studies, for instance, acoustophoresis^18–23^, magnetophoresis^24–29^, thermophoresis^30,31^, dielectrophoresis^32,33^, and electrophoresis^34^, have been carried out on active microfluidic separation technology.

These techniques offered precise control and separation capabilities, making them suitable for a wide range of applications. Due to the inherent electronegativity of microplastic particles, electrophoretic separation technology utilizing the ion concentration polarization effect has a natural advantage in separating charged particles. Voltage is  applied in microchannels embedded with ion-selective membranes or nanochannels, and ion depletion and ion enrichment regions are  created. This phenomenon is known as ion concentration polarization^35^. The formation of depletion and enrichment regions leads to a change in the electric field gradient within the microfluidics chip. This effect is  utilized to manipulate the movement of charged microparticles. Because of the advantage of this microfluidic technology in separating charged particles, it provides new approaches for seawater desalination^36^ and ion separation^37^. Previous works of numerous studies have focused on exploring influencing factors^38,39^ and theoretical analysis^40^. This microfluidic technique has greatly improved the effectiveness of separation and enrichment. On the contrary, the microchannel met many challenges such as manufacturing nanochannels, clogging of ion-selective membranes, and the limited lifespan of channels.

The researchers began to find new solutions to solve the above problems. In a major advance in 2008, Hlushkou et al.^41^ creatively proposed an electrochemical variant of the ion concentration polarization microchannel. This method used metal conductors in the microchannel instead of ion-selective membranes. When a driving voltage was applied, cathodic and anodic regions were induced on both sides of the metal conductor in this channel. This electrode was referred to as a bipolar electrode. Spontaneous redox reactions occurred on both sides of the cathode and anode, leading to different ion depletion and enrichment regions. It altered the movement trajectory of microplastic particles. Since this effect was generated through the Faraday reaction, this phenomenon was known as the Faradaic ion concentration polarization effect. It reduced the complexity of microchannel fabrication and addressed membrane clogging issues. More recent evidence^42,43^ showed that bipolar electrodes attracted widespread attention from researchers. Eden et al.^44^ used numerical simulation techniques to establish a comprehensive model of a bipolar electrode nanochannel. The model demonstrated that upon applying a sufficiently large voltage, the depletion zone spreads outward from the bipolar electrode. The control steps of the electrode reaction transitioned from electron transfer to mass transfer. Davies et al.^45^ designed a novel three-pronged channel for separating polystyrene microparticles with different electrophoretic mobilities. However, an additional buffer solution needed to be added to the microchannel solution to meet the conditions of ion concentration polarization. It caused secondary pollution to the water environment. Therefore, this method is only at the experimental stage and still has limitations in practical applications. To solve this problem, Thompson et al.^46^ introduced a separation channel model for separating plastic microbeads in an unbuffered solution. The feasibility of separating microplastics using this model was experimentally validated and verified through simulations. This method does not require the addition of any buffer solution to the microchannel, relying solely on the electric field gradient generated by the ions in the solution to separate microplastics. This approach was more practical and provided rich theoretical guidance for subsequent practical applications.

Research on the bipolar electrode ion concentration polarization principle is still in the experimental stage. There is a lack of sufficient theoretical studies and a comprehensive understanding of the influencing factors of this effect. The development of a sound theoretical framework requires the establishment of simulation models. Consequently, this article aims to: (a) propose a performance evaluation scheme for separating microplastic particles under unbuffered solution conditions. This paper focuses on how to build up the mathematical model of bipolar electrode microchannel and; (b) analyze the factors affecting the efficiency of microplastic separation by varying the structural and physical parameters within the microchannel. After analyzing the influencing factors, the optimal solution for separating microplastic particles from bipolar electrode bifurcation channels was found. Section “Principles of microplastic separation” describes the fundamentals of particle separation affected by bipolar electrodes in the channel. The governing equations describing the microfluidic problem are given. Section “Characteristics of bipolar electrodes” presents a simplification method of the microchannel model and a numerical simulation method of the separation channel. Section “Particle separation in the channel” presents the analysis of the influence factors of separation efficiency, and explains the mechanism of each factor. Section “Conclusions” discusses the relevant characteristics of the model and presents the conclusions of this study.

## Principles of microplastic separation

### Fundamental of microchannel

**Figure 1:**
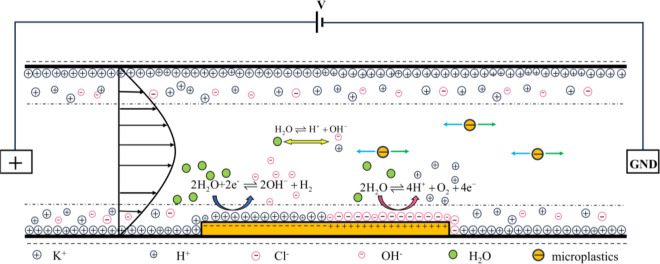
Controlling microplastic particle movement through bipolar electrode reaction.

The method of separating particles in an unbuffered solution can achieve the separation of microplastic particles without the addition of other solutions. This method can prevent secondary pollution in the aquatic environment in practical applications. The method offers advantages such as low cost and long lifespan, providing a more feasible solution for the separation of microplastic particles. The separation principle of microplastic particles is illustrated in Fig. 1.

When the fluid flows through the microchannel, the microchannel walls carry a negative charge. The ions in the diffusion layer undergo directed movement and the conductor produces induced charges to maintain the same potential within the conductor after an electric voltage *V* is applied. Due to the viscosity of the fluid, the ions induce electroosmotic flow. Since the diffusion layer has a local positive charge, the direction of the electroosmotic flow is the same as the movement direction of the cations.

**Figure 2:**
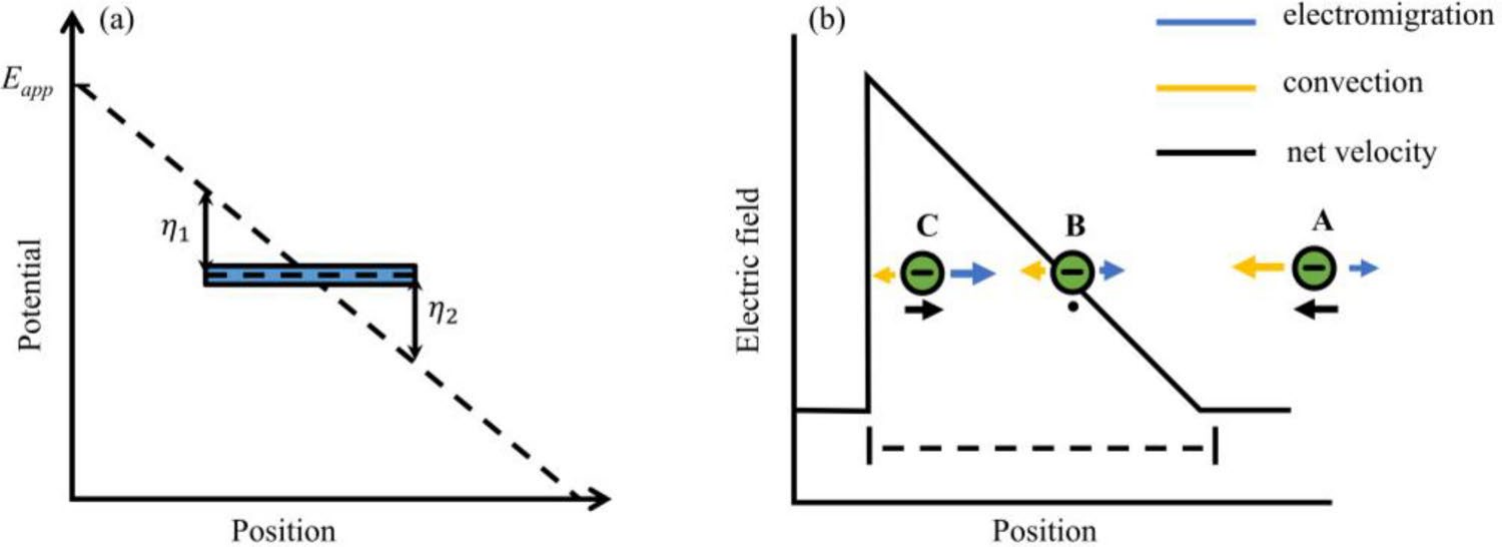
Distribution of electric potential and electric field near bipolar electrodes. (**a**) Solution potential and bipolar electrode potential. (**b**) Changes in electric field strength near the bipolar electrode and force acting on microplastic particles.

The potential near the conductor is illustrated in Fig. 2a. At the interface of the bipolar electrode, the potential difference drives redox reactions. The redox reaction involves water electrolysis under unbuffered conditions, producing H^+^ at the anode and OH^−^ at the cathode. The generation of ions creates a significant gradient change in electric field strength near the bipolar electrode in the solution. This change causes a redirection of microplastic particles at this location, achieving the separation effect.

The electric field strength near the cathode of the bipolar electrode is illustrated in Fig. 2b. Under a zero-pressure gradient flow, negatively charged microplastic particles are influenced by the opposing effects of electroosmotic flow and electromigration. The electroosmotic flow effect is dominant at position A, causing the microplastic particle to move leftward. Conversely, when the particles are at position C, these cause the microplastic particle to move rightward. Particles eventually reach position B where electrophoresis and electroosmotic flow reach a balance.

### Governing equation

The analysis of microchannel issues involves the coupling of multiple physical fields such as fluid flow, electrostatic field, and mass transfer. The corresponding equations for this are the Navier–Stokes equations, the Poisson equation, and the Nernst–Planck mass transfer equations. The Navier–Stokes equations are described as:

1 $$\rho (\frac{{\partial {\mathbf{u}}}}{\partial t} + {\mathbf{u}} \cdot \nabla {\mathbf{u}}) = \eta \nabla^{2} {\mathbf{u}} - \nabla p - \mathop \sum \limits_{i = 1}^{n} Fz_{i} c_{i} \nabla V$$

2 $$\nabla \cdot {\mathbf{u}} = 0$$

where **u** represents fluid velocity; *η* stands for fluid dynamic viscosity;* ρ* is the fluid density; *F* is the Faraday constant; *V* is the applied voltage across the microchannel;* z*_*i*_ and* c*_*i*_ represent the charge number and concentration of i species, respectively. Due to the simulation of microfluidic, fluid flows slowly and Reynolds number *Re* is small at the steady condition in the channel. Therefore, Eq. (1) simplifies to the following form:

3 $$\eta \nabla^{2} {\mathbf{u}} - \nabla p - \mathop \sum \limits_{i = 1}^{n} Fz_{i} c_{i} \nabla V = 0$$

We use the Poisson equation to solve for electrostatic fields, which is described as follows:

4 $$\nabla^{2} V = - \frac{\rho }{\varepsilon }$$

where *ε* is the relative permittivity; *ρ* is the space charge density in the solution, which is $\rho = \sum\limits_{i = 1}^{n} {Fz_{i} c_{i} }$; due to the channel in the micrometer or millimeter range, the electric double layer has little influence on the solution. therefore, we can assume the electrical neutrality in the solution. The Poisson equation can simplify the Laplace equation:

5 $$\nabla^{2} V = 0$$

We utilize the Nernst-Planck equation to solve concentration distribution in the Runners:

6 $$\frac{{\partial c_{i} }}{\partial t} + \nabla \cdot {\mathbf{J}}_{i} + {\mathbf{u}} \cdot \nabla c_{i} = R_{i}$$

7 $${\mathbf{J}}_{i} = - D_{i} \nabla c_{i} - z_{i} u_{m,i} Fc_{i} \nabla V$$

where *D*_*i*_ represents the diffusion coefficient of i species, respectively; **J**_*i*_ represents the flux of i species, *u*_*m,i*_ is the electrophoretic mobility of i species; *R*_*i*_ stands for the reaction source term for i species. In the unbuffered solution, we consider the reaction source term arising from the electrolysis of water. The equation of the chemical reaction is shown below:

$${\text{H}}_{{2}} {\text{O}} \rightleftharpoons {\text{H}}^{ + } {\text{ + OH}}^{ - }$$

the reaction source terms *R* for H^+^ and OH^−^ in Eq. (6) can be determined through reaction rates. The equations are expressed as:

8 $$R_{{[{\text{OH}}^{ - } ]}} = k_{f} [{\text{H}}_{{2}} {\text{O}}] - k_{b} [{\text{OH}}^{ - } ][{\text{H}}^{ + } ]$$

9 $$R_{{[{\text{H}}^{ + } ]}} = k_{f} [{\text{H}}_{{2}} {\text{O}}] - k_{b} [{\text{OH}}^{ - } ][{\text{H}}^{ + } ]$$

where *R*_[OH_-_]_ and *R*_[H_^+^_]_ represent the chemical reaction rates for OH^−^ and H^+^, respectively. *k*_*f*_ and *k*_*b*_ are the forward and reverse reaction rate constants for the electrode reaction.

## Characteristics of bipolar electrodes

### Model simplification

The intricate nature of solving differential equations involves strongly coupled multi-physics phenomena in numerical simulations of microfluidics. This model often entails challenging convergence and computational difficulties. We choose to judiciously simplify the microchannel model before embarking on the numerical simulations.

**Figure 3:**
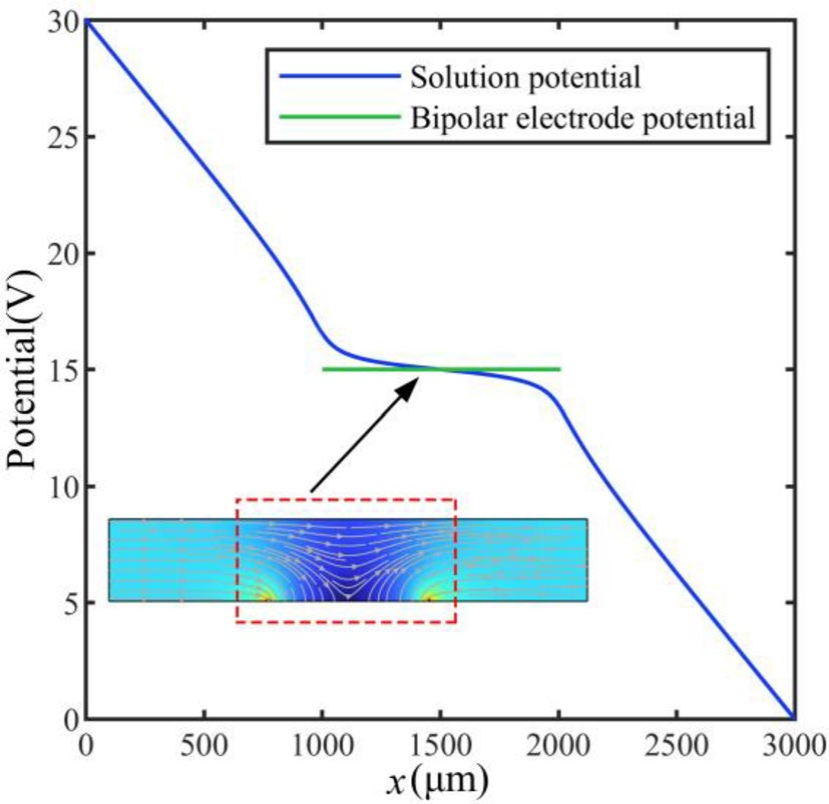
Electric field and potential distribution within the microchannel.

The electric field and potential generated by the bipolar electrode reaction is shown in Fig. 3. The electric field varies the most at the ends of the bipolar electrode and changes relatively slowly at other positions of the bipolar electrode^47^. The potential in Fig. 3 shows that the overpotential is highest at positions adjacent to the bipolar electrodes.

**Figure 4:**
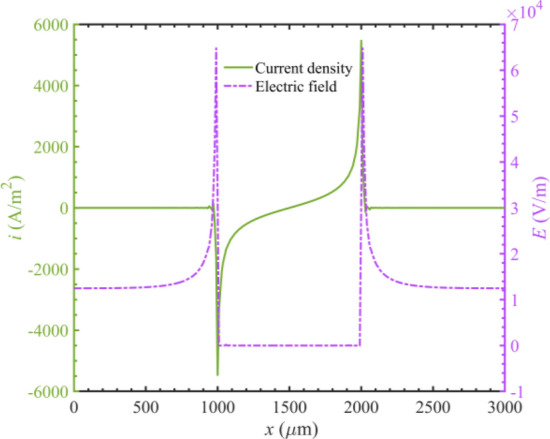
Current density and electric field distribution within the microchannel.

The electric field and current density distribution within the microchannel are depicted in Fig. 4. These results show that the electric field is highest at positions adjacent to the bipolar electrodes, and the current density induced by the reaction is also maximized corresponding to the locations. It can be seen that the oxidation–reduction reaction occurs most intensely at the ends of the bipolar electrode from these results. Therefore, we model the ends of the bipolar electrode to replace the entire bipolar electrode, which can reduce model complexity.

**Figure 5:**
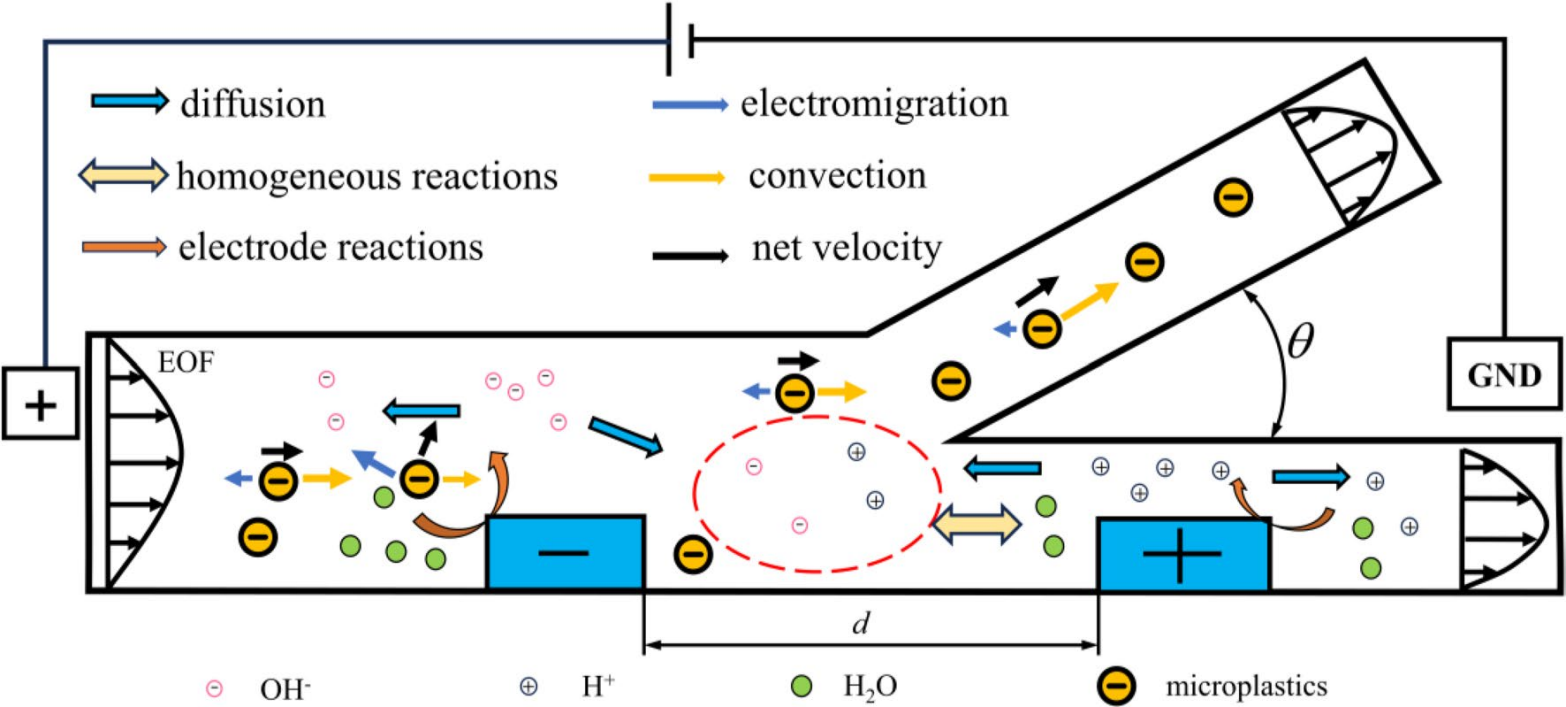
The separation principle in the bifurcation channel.

The structure of the separation channel is shown in Fig. 5. The OH^−^ generated by the cathodic reaction increases the conductivity near the cathode. The electric field gradient is changed by increased conductivity, which alters the magnitude and direction of the electric migration. As a result, the plastic particles flow out of the upper channel to achieve the separation effect. The influence of parameters such as the applied voltage, the separation channel angle, and the distance of the bipolar electrode on the separation efficiency of plastic microbeads is investigated.

### Boundary conditions

Concerning the schematic diagram of the bifurcated channel in Fig. 5, the main boundary conditions for this system are the surfaces of the bipolar electrodes. The oxidation reactions occur at the anode of the bipolar electrode producing H^+^. The electrode reaction equation is shown below:

$${2} {\text{H}}_{{2}} {\text{O}} \rightleftharpoons {4} {\text{H}}^{ + } {\text{ + O}}_{{2}} {\text{ + 4e}}^{ - }$$

The current density generated at the anode reaction obeys the Butler–Volmer equation shown below^48^:

10 $$i_{loc} = i_{0,a} \left[ {\exp \left( {\frac{{\alpha \eta_{a} }}{RT}} \right) - \exp \left( {\frac{{ - \beta \eta_{a} }}{RT}} \right)} \right]$$

11 $$\eta_{a} = \phi_{s} - \phi_{l} - E_{eq,a}$$

In Eqs. (10) and (11), *i*_*loc*_ represents the local current density for the anodic reaction; *i*_*0*_ denotes the exchange current density for the anodic reaction; *α* and *β* are the transfer coefficients for the anodic and cathodic reactions, respectively; *R* is the universal gas constant; *T* stands for temperature; *η*_*a*_ denotes the overpotential at the anode of the bipolar electrode; *ϕ*_*s*_ represents the potential of electrode and *ϕ*_*l*_ is the potential of the solution near the bipolar electrode;* E*_*eq,a*_ represents the electrode potential at the temperature *T* solved by Nernst equation. The equation is shown below:

12 $$E_{eq,a} = E_{0,a} - \frac{RT}{{nF}}\ln \left( {\frac{{C_{{[{\text{H}}^{ + } ]}} }}{{C_{{0,[{\text{H}}^{ + } ]}} }}} \right)$$

In Eq. (12), *E*_*0,a*_ represents the standard electrode potential for the anodic reaction; *F* is the Faraday constant; *n* is the number of electrons involved in the reaction transfer; ${C}_{[\text{H}^{+}]}$ is the surface H^+^ concentration at the bipolar electrode, and ${C}_{0,{[\text{H}^{+}]}}$ is the H^+^ concentration in the bulk solution.

The rate of chemical reaction of the H^+^ follows Faraday's law:

13 $$R_{{[{\text{H}}^{ + } ]}} = \frac{{v_{{[{\text{H}}^{ + } ]}} i_{loc} }}{nF}$$

In Eq. (13), ${v}_{{[\text{H}^{+}]}}$ represents the stoichiometric number of H^+^ in the anodic reaction.

The chemical reaction equation for producing OH^−^ at the cathode of the bipolar electrode is as follows:

$$2 {\text{H}}_{2} {\text{O + 2}} {\text{e}}^{ - } \rightleftharpoons 2 {\text{OH}}^{ - } + {\text{H}}_{2}$$

The cathodic reaction at the bipolar electrode also follows the Butler-Volmer equation and Faraday's law:

14 $$i_{loc} = i_{0,c} \left[ {\exp \left( {\frac{{\alpha \eta_{c} }}{RT}} \right) - \exp \left( {\frac{{ - \beta \eta_{c} }}{RT}} \right)} \right]$$

15 $$\eta_{c} = \phi_{s} - \phi_{l} - E_{eq,c}$$

16 $$E_{eq,c} = E_{0,c} - \frac{RT}{{nF}}\ln \left( {\frac{{C_{{[{\text{OH}}^{ - } ]}} }}{{C_{{0,[{\text{OH}}^{ - } ]}} }}} \right)$$

17 $$R_{{[{\text{OH}}^{ - } ]}} = \frac{{v_{{[{\text{OH}}^{ - } ]}} i_{loc} }}{nF}$$

where *i*_*0,c*_ represents the exchange current density for the cathodic reaction, *η*_*c*_ is the overpotential for the cathodic reaction; *E*_*eq,c*_ is the electrode potential for the cathodic reaction at the temperature *T*; *E*_*0,c*_ is the standard electrode potential for the cathodic reaction; $C_{{[{\text{OH}}^{ - } ]}}$ and $C_{{0,[{\text{OH}}^{ - } ]}}$ represent the OH^−^ concentration near the cathodic electrode and in the bulk solution, respectively; *n* is the stoichiometric number of OH^−^.

The boundary conditions applied at the inlet of the microchannel: (1) specified ion concentration *c*_0*,i*_; (2) specified voltage *V*_*0*_; and (3) pressure is set to 0:

18 $$c = c_{0,i} ,\;\;\; V = V_{0} ,\;\;\; p = 0$$

The boundary conditions applied at the outlet of the microchannel: (1) zero diffusion flux, (2) zero voltage, and (3) pressure is set to 0:

19 $${\mathbf{n}} \cdot D_{i} \nabla c_{i} = 0,\;\;\; V = 0,\;\;\; p = 0$$

The boundary conditions imposed at the microchannel walls: (1) zero flux, (2) electrical insulation, and (3) the velocity of electroosmotic flow within the microchannel follows the Helmholtz–Smoluchowski equation^49^:

20 $$- {\mathbf{n}} \cdot {\mathbf{J}}_{{\mathbf{i}}} = 0,\;\;\; {\mathbf{n}} \cdot {\mathbf{D}} = 0,\;\;\; {\mathbf{u}} = - \frac{{\zeta \varepsilon {\mathbf{E}}}}{\eta }$$

where *ζ* represents the potential difference between the diffusion layer and the bulk solution, *E* is the electrical field strength inside the microchannel, *E* = *− *∇*V*, and *V* is the applied potential within the solution.

### Model verification

We use the straight channel model commonly used in the field of microfluidics to verify the feasibility of the simulation method to ensure that the simulation results are real and valid. The results of the simulations are compared with the experimental results of other researchers to prove that the simulation method is feasible.

**Figure 6:**
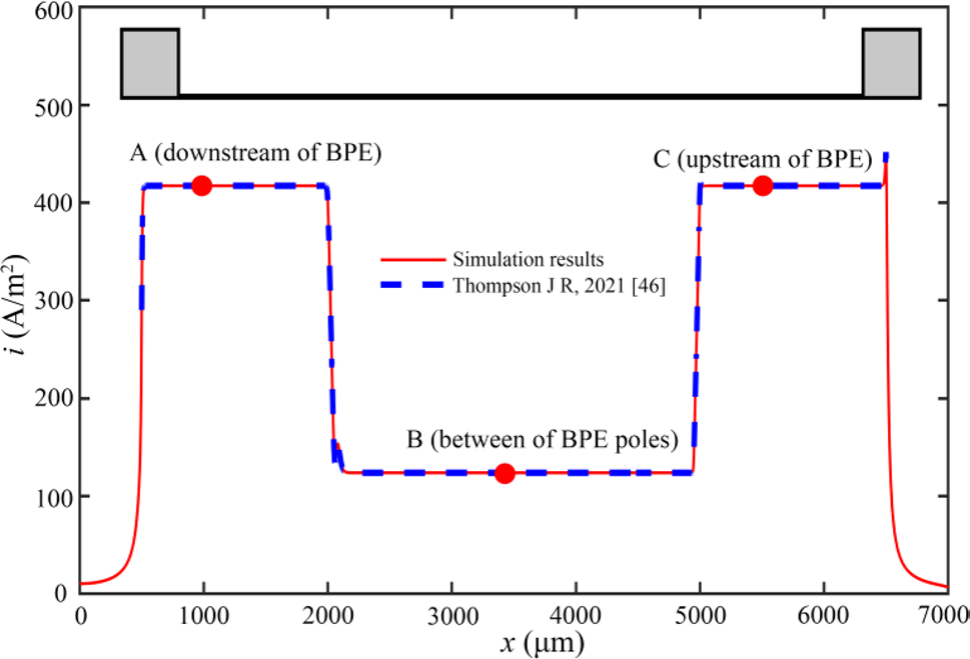
Ionic current density distribution.

Ionic current density is depicted in Fig. 6. Because of the electrode reaction of the model, the result of ionic current density can vary along the length of the channel. These variables changed most significantly around the bipolar electrodes. Therefore, the values of current density near the three bipolar electrodes are extracted and compared with the experimental data. The results of the simulation show an almost equal effect to those of other researchers^46^, which means a correct simulation model.

The ionic current density is measured at three positions in the experiments of other scientific researchers, which are 500 μm of the electrode cathode (upstream of BPE), 1500 μm downstream of the electrode cathode (between BPE poles), and 1000 μm downstream of electrode anode (downstream of BPE). These results are shown in Table 1. The results show that the simulation results are not much different from the experimental data within the allowable range of error, and the results obtained by the simulation model are valid.

**Table 1:** Ionic current density (unit: A/m^2^).

	Position A	Position B	Position C
Simulation	417.29	123.96	417.29
^46^	410 ± 30	110 ± 40	410 ± 30

### Particle separation in the channel

The separation microchannel structure is shown in Fig. 5. A solution of KCl with a concentration of 5 mol/m^3^ (${z}_{{\text{K}^{+}}}$ = 1,  ${z}_{{\text{cl}^{-}}}$ = − 1,  ${z}_{{\text{H}^{+}}}$ = 1,  ${z}_{{\text{OH}^{-}}}$ = − 1,  ${D}_{{\text{K}^{+}}}$ = 1.97 × 10^–9^ m^2^/s,  ${D}_{{\text{cl}^{-}}}$ = 2.033 × 10^–9^ m^2^/s,  ${D}_{{\text{H}^{+}}}$ = 9.103 × 10^–9^ m^2^/s,  ${D}_{{\text{OH}^{-}}}$ = 5.28 × 10^–9^ m^2^/s) is used at the inlet of the channel. In order to ensure the validity of the model and to be able to implement the simulation function, we assume microplastics are evenly distributed in solution. A polystyrene microplastic particle is used and its concentration is 3 × 10^–12^ mol/m^3^. Although environmental factors lead to different charges of microplastic particles, we choose a special kind of polystyrene microplastic, whose charge is − 2 (*z*_Bead_ = − 2). Microbeads can be affected by diffusion in this simulation, so the diffusion coefficient is considered, which is 7.85 × 10^–8^ m^2^/s (*D*_Bead_ = 7.85 × 10^–8^ m^2^/s). Additionally, the diameter of particles is 0.99 μm. The relative permittivity of the solution is 80, and the zeta potential is − 80 mV. The governing Eqs. (2), (3), (5), (6) and (7) are solved using the commercial finite element software Comsol Multiphysics V6.0. By varying the applied voltage *V*_*0*_, separation channel angle *θ*, and position of the bipolar electrode *d*, the microfluidics separation mechanism and the impact on the efficiency of microchannel separation are investigated.

**Figure 7:**
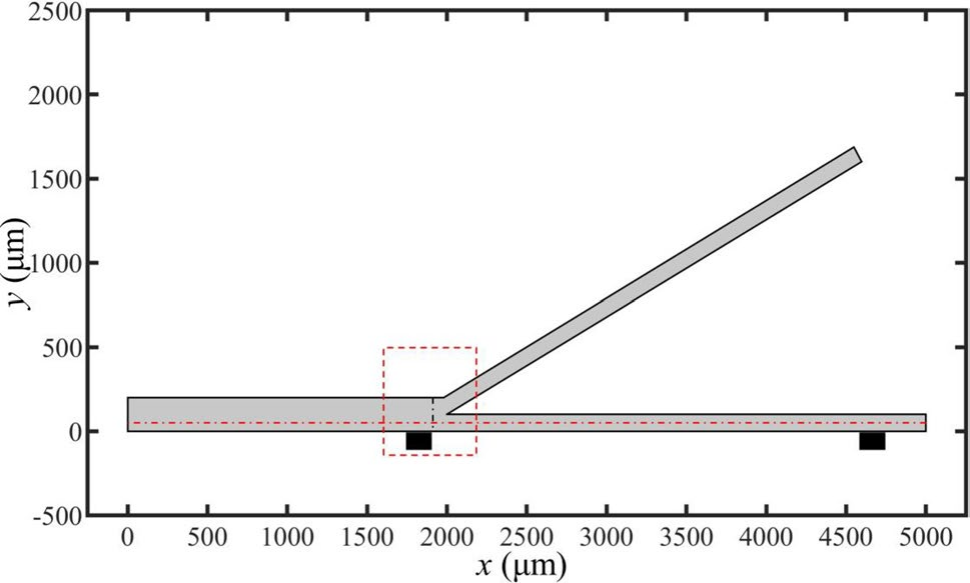
Numerical simulation geometric model of microchannel.

The geometry of the used microfluidic channel is depicted in Fig. 7. Upon applying a voltage *V*_*0*_ on both sides of the separation channel, the bipolar electrodes at the bottom of the channel become activated. Microplastic particles flow out from the top of the channel, while purified water flows out from the bottom of the channel.

### Effect of applied voltage on separation efficiency

When the applied voltage *V*_*0*_ is varied, the magnitude of the overpotential on the bipolar electrode is changed, which affects the oxidation–reduction reaction rate. Finally, the reaction rate impacts the separation efficiency of microparticles.

**Figure 8:**
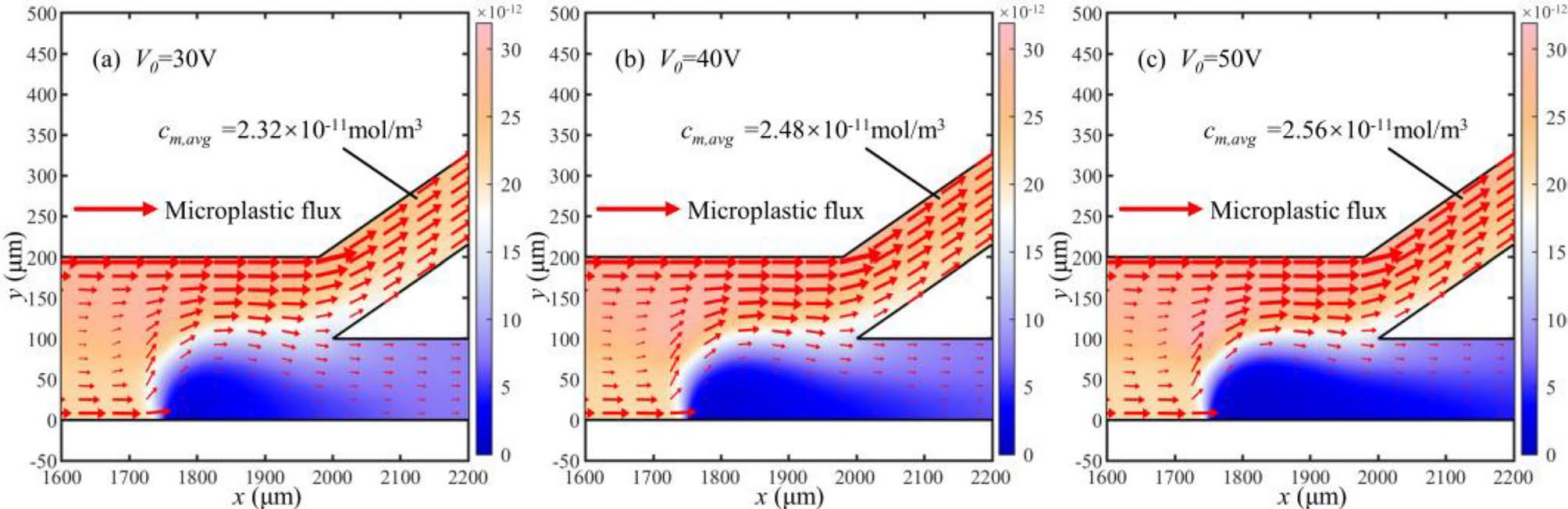
Concentration and flux distribution of microplastic particles in partial microchannels at different voltages (unit: mol/m^3^).

The concentration distribution of microplastic particles is illustrated in a partial model of the bifurcation channel in Fig. 8, indicated by the red dashed box in Fig. 7. *c*_*m,avg*_ is the average concentration of microplastics in the top channel. The electrochemical reaction rate near the cathode accelerates as the voltage *V*_*0*_ increases, which leads to a change in the nearby electric field strength. This alteration impacts the force experienced by microplastic particles in the solution and results in a decrease in particle concentration in the bottom channel and an increase in concentration in the top channel.

**Figure 9:**
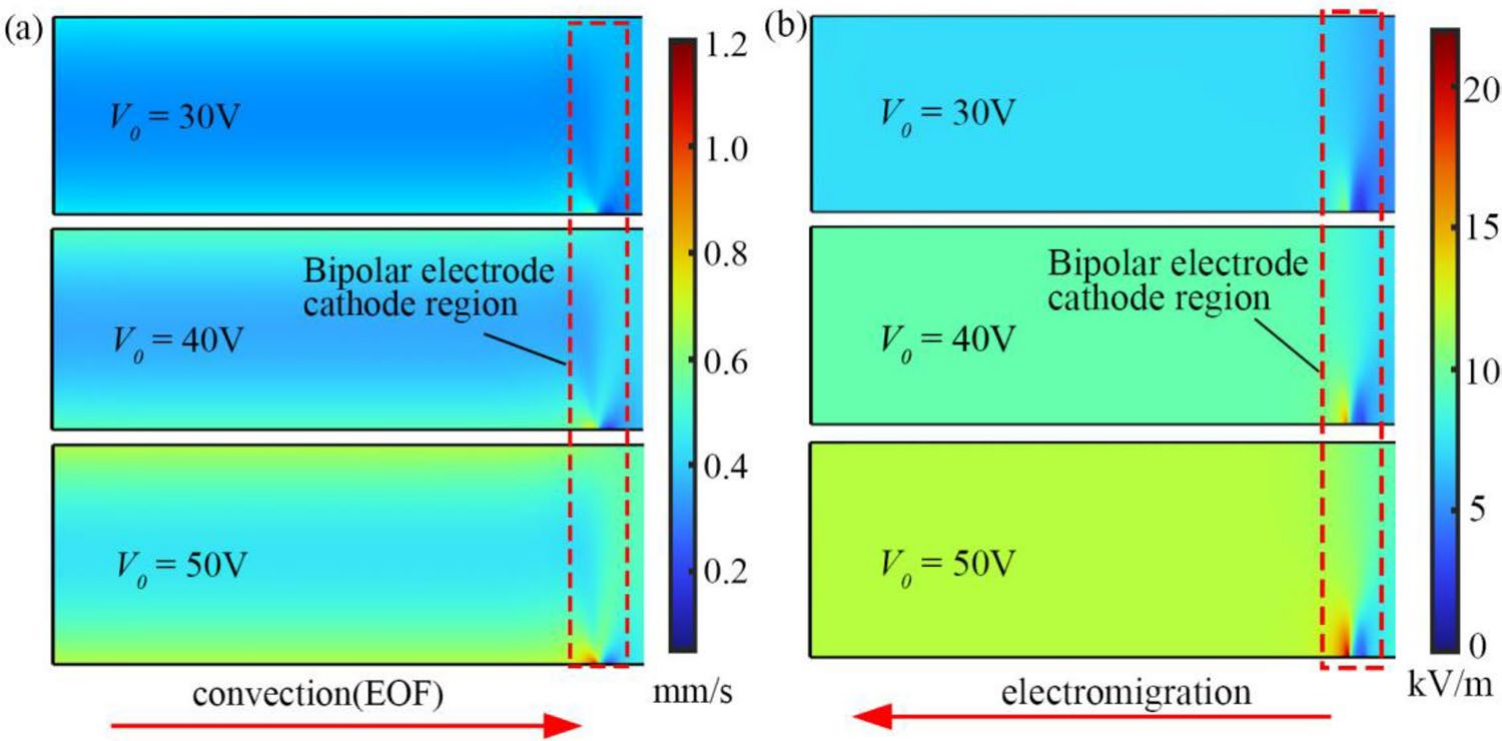
Distribution of flow and electric fields at different voltages. (**a**) Velocity distribution in the microchannel (unit: mm/s). (**b**) Electric fields distribution in the microchannel (unit: kV/m).

We simulated the distribution of flow and electric fields to consider the convection and electromigration of microplastics. The flow rate of electroosmotic flow is accelerating with the increase of the applied voltage *V*_*0*_, which is depicted in Fig. 9a. Electroosmotic flow is predominant in the motion of particles away from the bipolar electrode. Additionally, the properties of fluid flow become complex near the electrode for the changing electric field.

We can also observe the changing electric field in Fig. 9b. The reaction of the electrode affects the electric field in the bipolar electrode cathode region, which generates a large electric gradient. the direction and magnitude of electroosmotic flow and electromigration in this region, therefore, they can affect the motion trajectory of microplastics.

**Figure 10:**
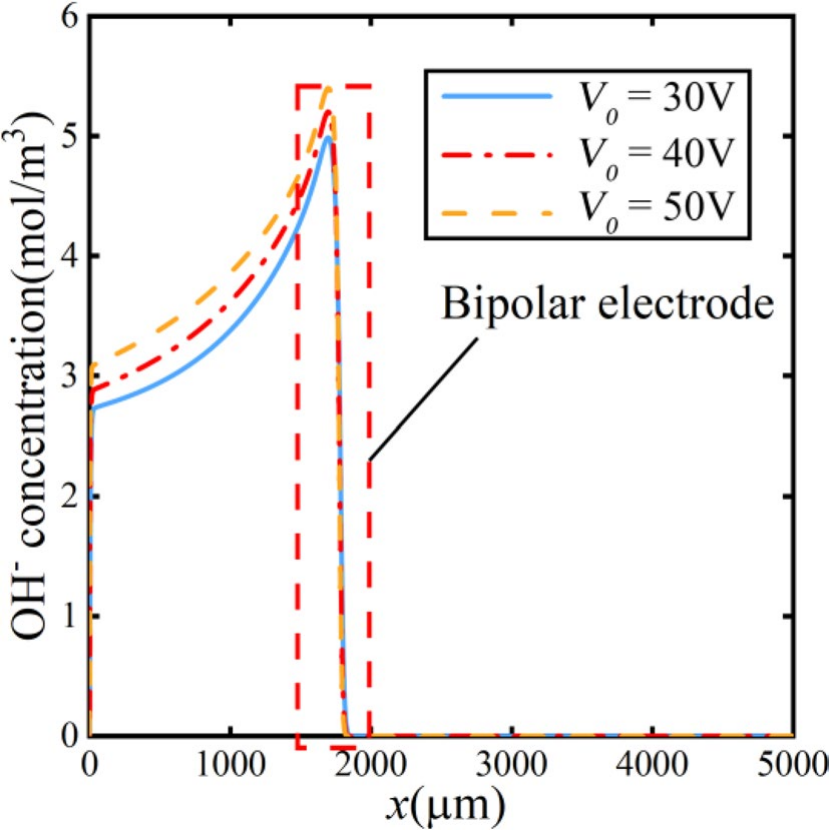
Concentration of hydroxide ions at different voltages.

To study the factors affecting the electric field, the simulation was conducted to model the ion concentrations at different voltages. Figure 10 illustrates the distribution of OH^−^ ion concentration along the centerline of the bottom channel as indicated by the red dashed line in the schematic of Fig. 7. With the increase in voltage, the concentration of hydroxide ions near the cathodic increases. The increased concentration indicates a faster chemical reaction rate at the bipolar electrode.

**Figure 11:**
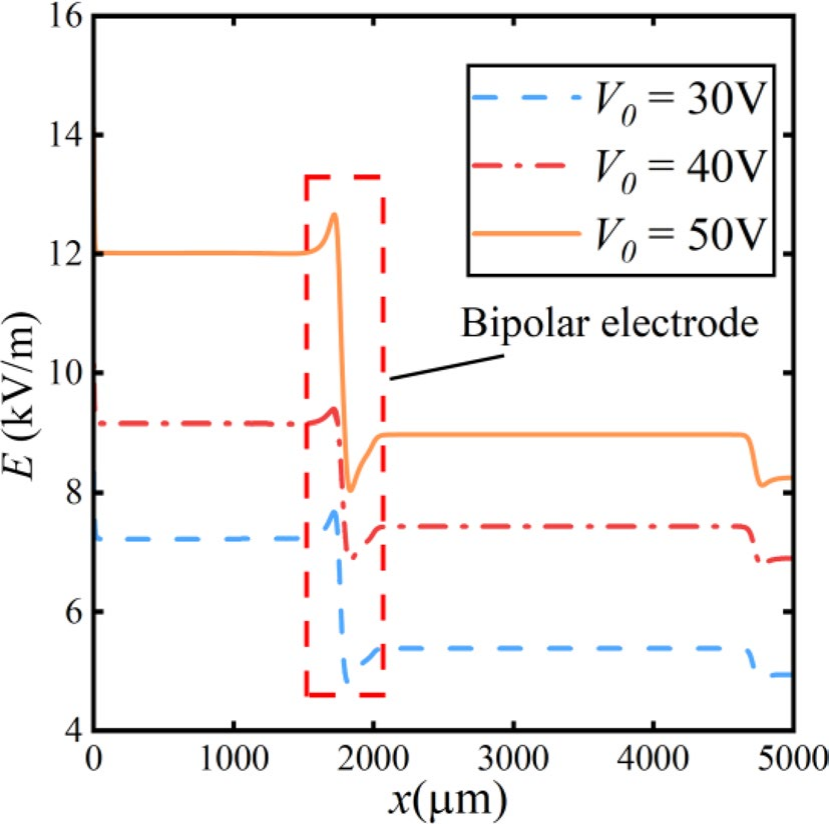
Electric field distribution at different voltages.

The distribution of the electric field at the same position is shown in Fig. 11. The change in the chemical reaction rate results in a greater variation of the electric field near the cathode. The varying electric field alters the trajectory of microplastic particles.

**Figure 12:**
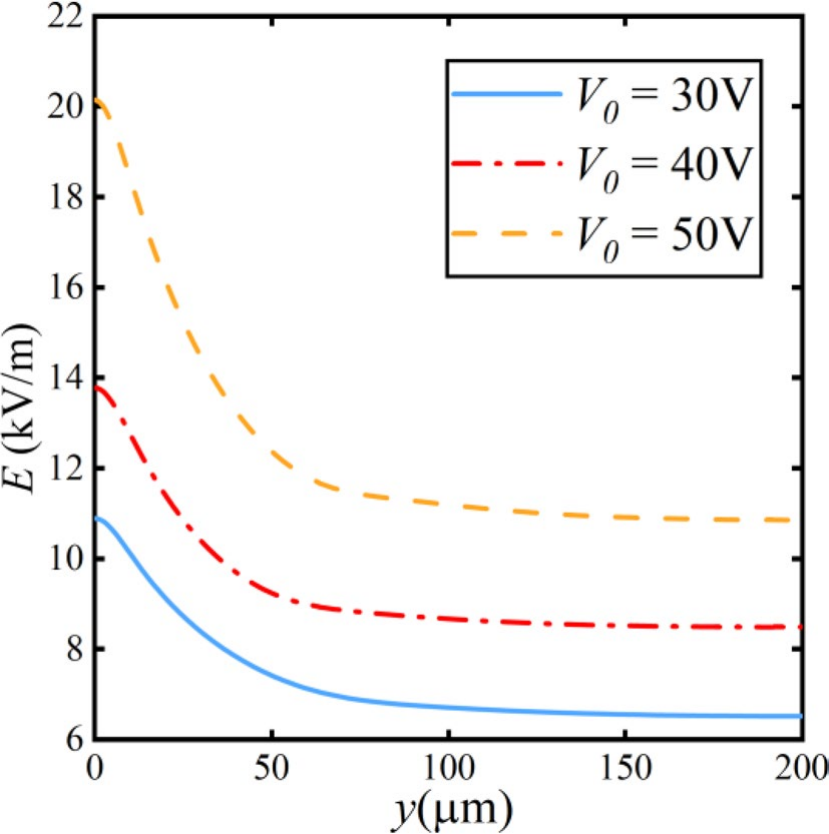
Electric field along the y-direction at different voltages.

The curves represent the electric field strength at *x* = 1900 μm in Fig. 12, as indicated by the black dashed line in the schematic of Fig. 7. With the increase in the applied voltage *V*_*0*_, the overall electric field near the cathode rises. This phenomenon enhances the electric migration effect experienced by microplastic. The initial equilibrium is disrupted because of the increasing electric migration effect. The microplastic particles move toward the lower electric field direction.

**Figure 13:**
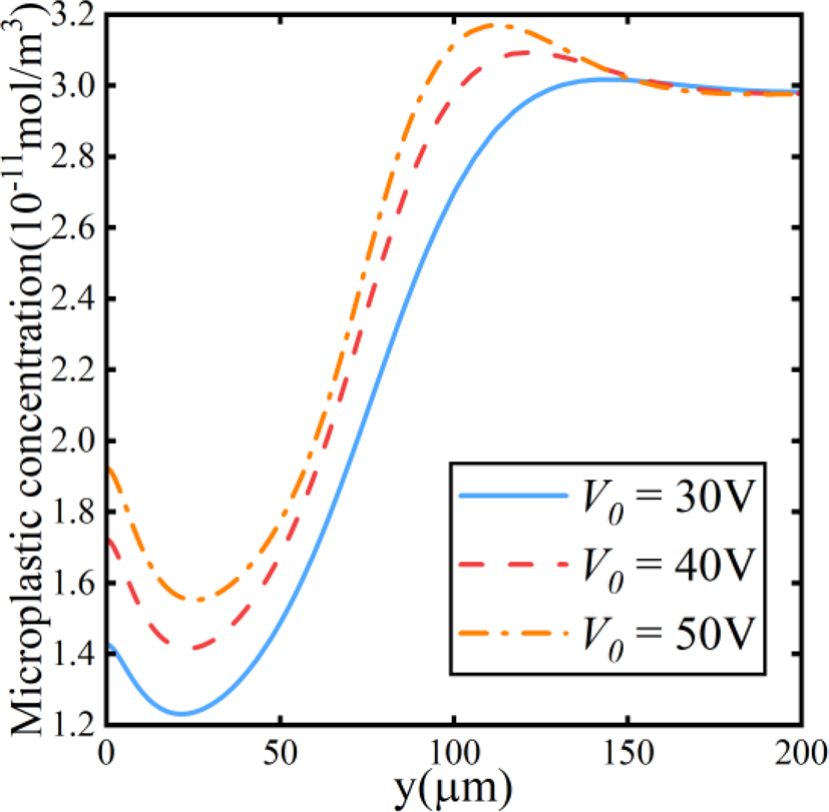
Microplastic particle concentration along the y-direction at different voltages.

Figure 13 depicts the distribution of microplastic concentration at the same location. Due to the alteration in electric field strength, the forces acting on microplastic particles are modified and influence the concentration of microparticles near the cathode. Lastly, the force affects the trajectory of the particles.

To investigate the influence of different voltages on the separation factor of plastic, we define the separation factor *r* as the ratio of the concentration of the tiny plastic in the top channel to that in the bottom channel.

21 $$r = \frac{{c_{m,up} }}{{c_{m,bottom} }}$$

*c*_*m,up*_ and *c*_*m,bottom*_ represent the microplastic particle concentration in the top channel and the bottom channel, respectively. A higher value of *r* implies a greater proportion of microplastic particles exiting from the top channel, which indicates a more effective separation. Conversely, a lower *r* implies poorer separation.

**Figure 14:**
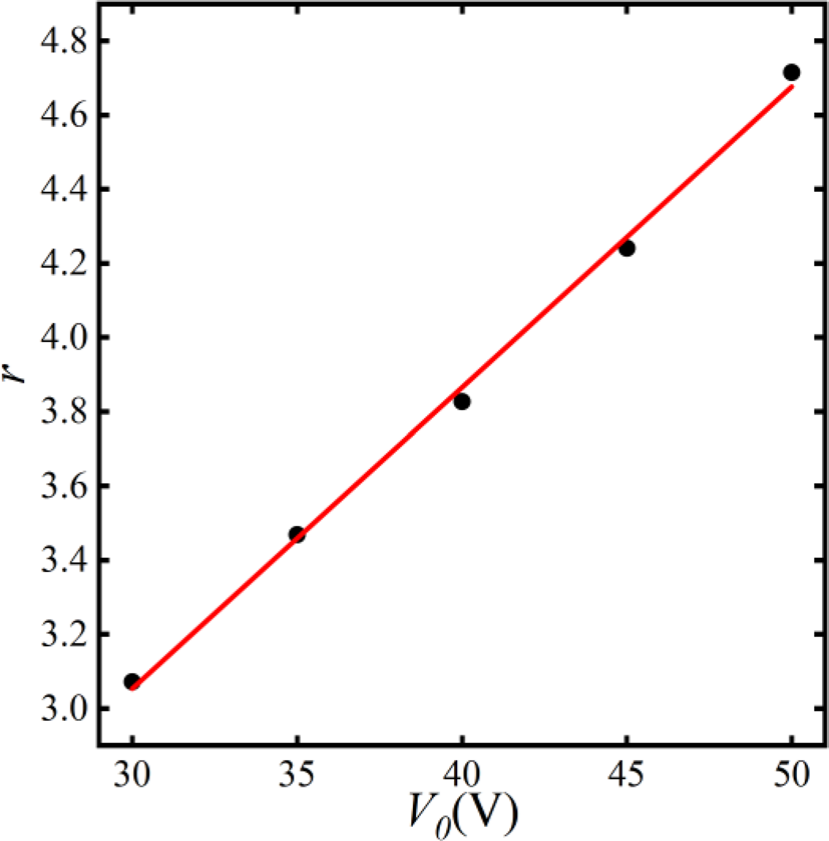
The relationship between separation factor and voltage variation.

The variation of the separation factor under different voltages is illustrated in Fig. 14. As the voltage *V*_*0*_ increased from 30 to 50 V, the separation factor increases by up to about 50%. The relationship between voltage and separation factor is as follows:

22 $$r = 0.62 + 0.081V_{0}$$

Due to the large electromigration effect of the accelerated reaction electrochemical reaction rate, this phenomenon indicates an improved separation efficiency with the development of voltage. However, with the increase in voltage, more secondary reactions occurred on the bipolar electrodes. These reactions can render the separation of microplastic particles uncontrollable. Simultaneously, escalating the voltage leads to an increase in the temperature within the microchannels because of the generation of more heat. Therefore, it is imperative to comprehensively consider the influence of voltage based on the actual application scenario.

### Effect of separation channel angle on separation efficiency

**Figure 15:**
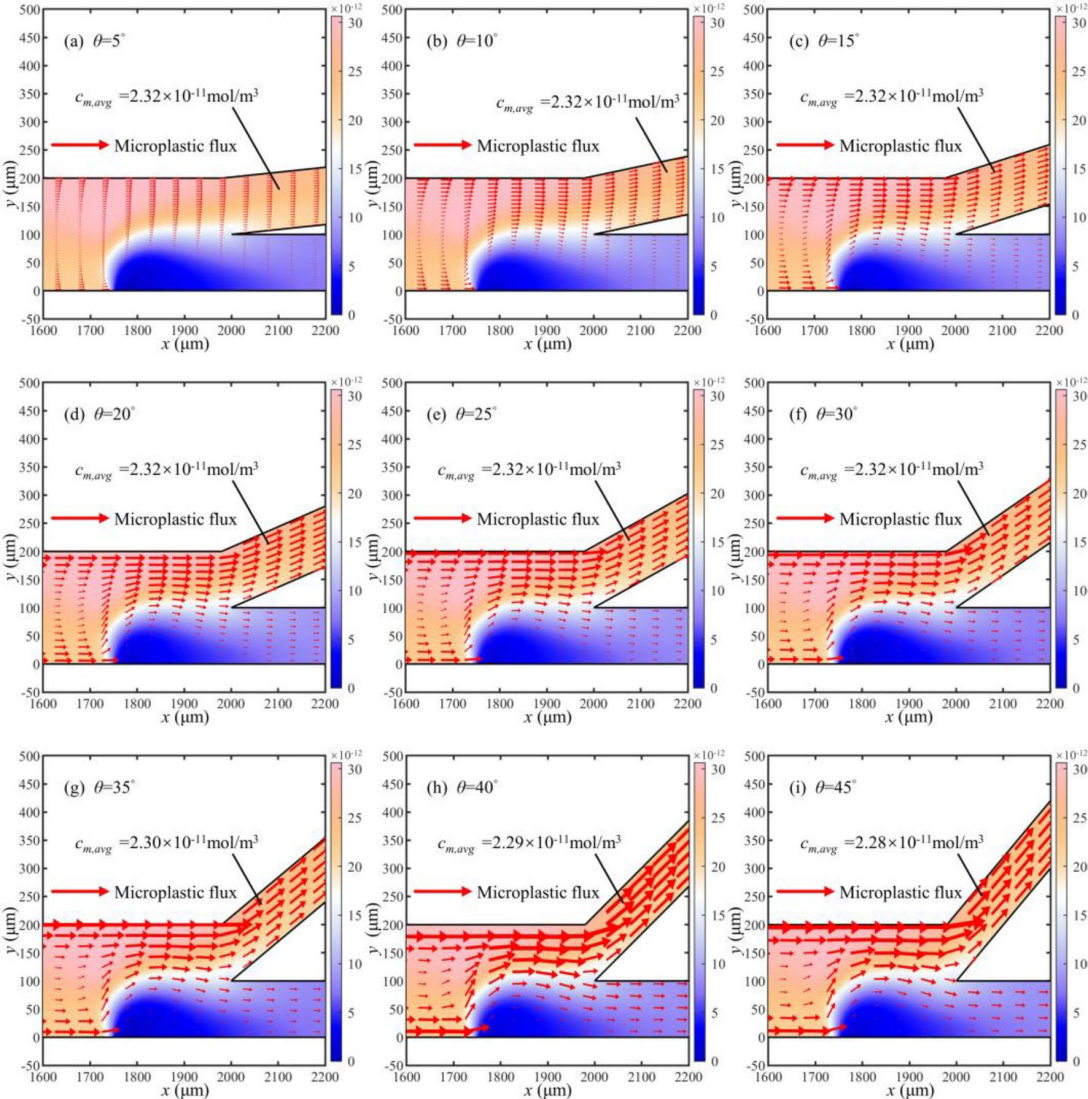
Concentration and flux distribution of microplastic particles in partial microchannels at different angles (unit: mol/m^3^).

The varying angle *θ* of the separation channel alters the flow patterns of the fluid, which causes a shift in the locations where H^+^ and OH^−^ accumulate. This change affects the magnitude of the electric and the trajectory of the microplastic particles field within the microchannel. The microplastic concentration and flux near the cathode are depicted in Fig. 15. The average concentration of the upper channel is almost constant, and the flux changes very little at angles between 5° and 30°. When the angle is greater than 30°, the flux of microplastic particles changes significantly.

**Figure 16:**
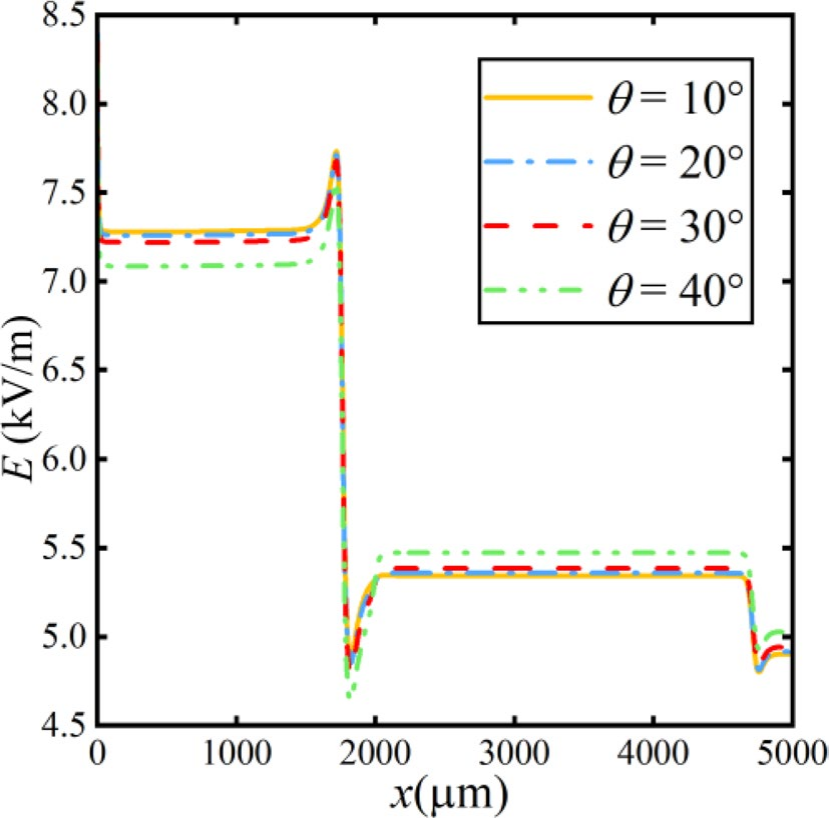
Distribution of electric field strength at different angles.

To further explore the above changes, we simulate the electric field inside the microchannel. The distribution of the electric field is illustrated along the midline of the bottom channel in Fig. 16. The conductivity of the solution changes very little because the overall concentration in the solution barely changes at angles between 5° and 30°. Therefore, the electric field does not change much in this range. However, the local electric field strength changes due to the change in particle flux at more than 30°.

**Figure 17:**
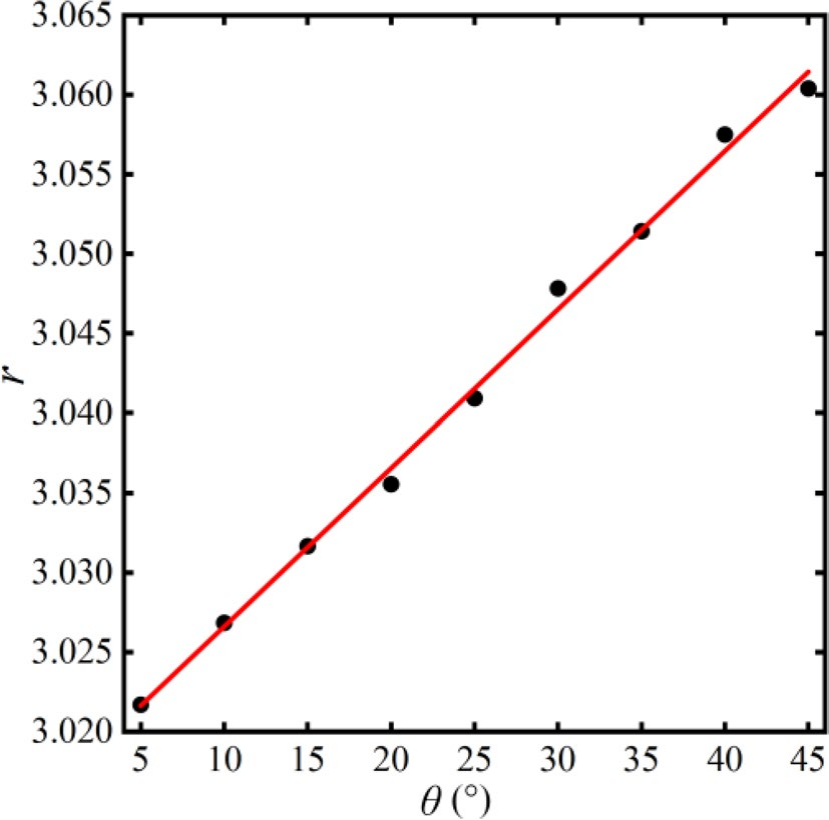
The relationship between separation factor and angle variation.

The relationship between the angle and the separation factor is depicted in Fig. 17. The relationship between angle and separation factor is as follows:

23 $$r{ = }3.017 + 0.001\theta$$

As the angle *θ* of the bifurcation channel continuously increases, the electric field strength remains relatively constant. Therefore, the magnitude of the separation factor ranges from 3.02 to 3.06, showing a subtle variation in the separation efficiency. It manifests that the varying angle has little effect on separation efficiency.

### Effect of bipolar electrode distance on separation efficiency

The cathode position of the bipolar electrode represents the most dynamically changing region of the electric field. In this simulation, we keep the position of the anode fixed while altering the distance *d* between the two electrodes to change the cathode position. Altering the cathode position leads to variations in the most dynamically changing region of electric field strength. Consequently, the electro-migration effect experienced by microplastic particles is altered and affects the trajectory of plastic microbead motion.

**Figure 18:**
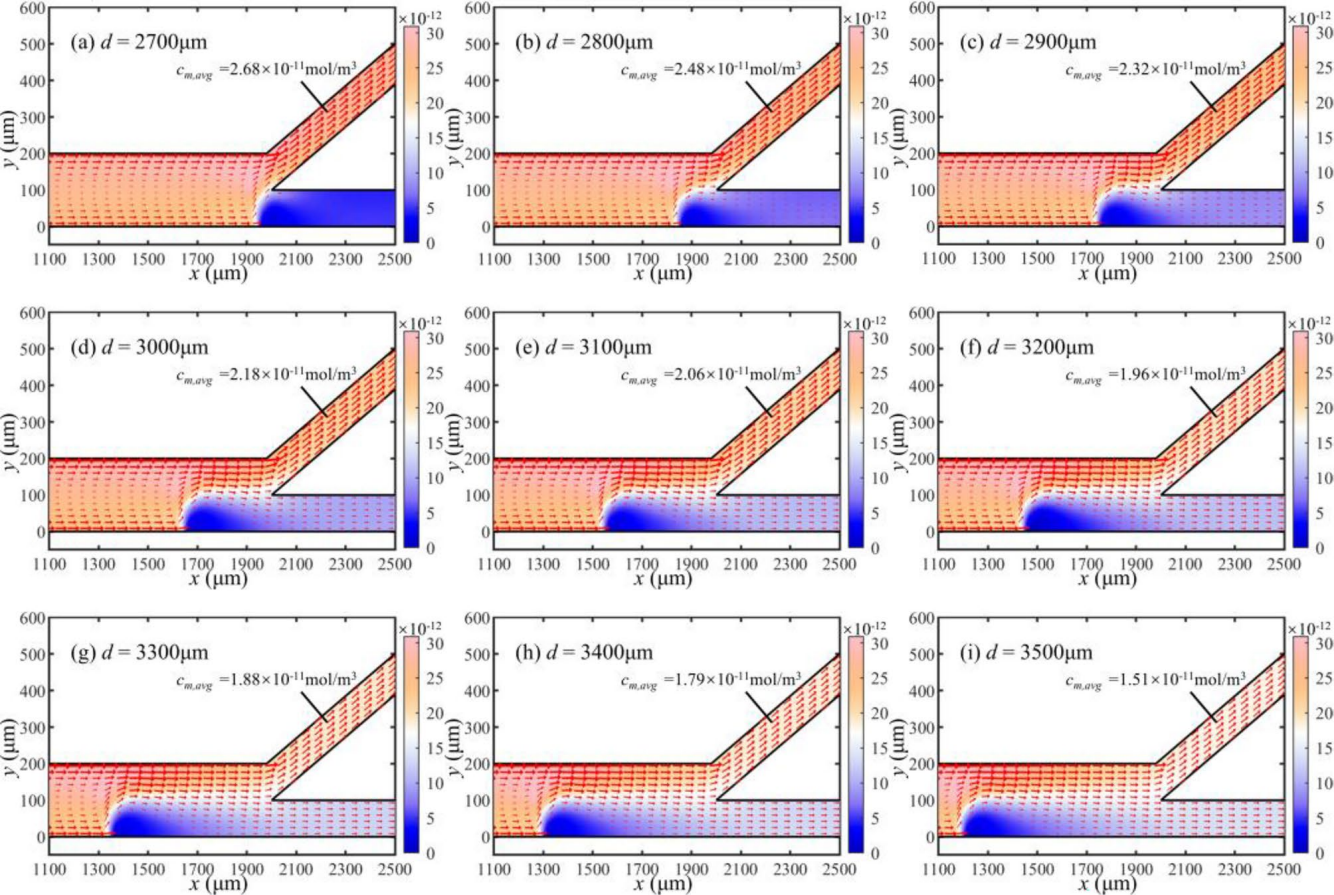
Concentration and flux distribution of microplastic particles in partial microchannels at different bipolar electrode distances (unit: mol/m^3^).

The concentration distribution of microplastic particles is presented in the channel for different cathode positions of the bipolar electrode in Fig. 18. Figure 18 shows that the concentration of microplastic particles rises in the bottom channel and decreases in the top channel as distance *d* between the two electrodes increases. The phenomenon indicates a weakening of the separation efficiency.

**Figure 19:**
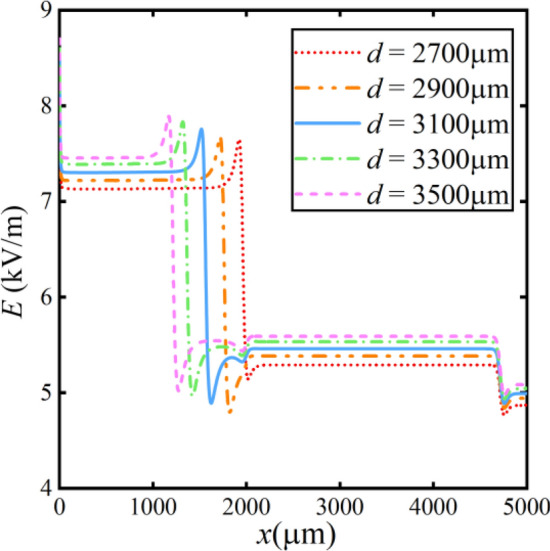
Distribution of electric field at different bipolar electrode distances.

To further analyze the results of the microplastic concentration distribution, we conduct additional simulations involving electric field and microplastic particle concentration. Figure 19 shows the electric field at the centerline position of the bottom channel.

It is evident that the electric field near the bipolar electrode increases significantly from 7.5 to 7.9 kV/m when the cathode of the bipolar electrode moves towards the positive voltage side. This increase is attributed to an increased potential near the inlet of the runners. The rate of chemistry reaction is increased by elevated potential, which leads to increased solution conductivity. Significant variations in the electric field gradient occur near the bipolar electrode.

However, due to the premature change in the electric field gradient within the microchannel, microplastic particle concentrations become re-mixed after separation. It fails to achieve the desired separation effect. When the bipolar electrode is close to the bifurcation point of the channel (*x* = 2000 μm), the electric field is the highest near the bifurcation point. Microplastic particles experience a substantial electro-migration effect, resulting in more thorough separation.

**Figure 20:**
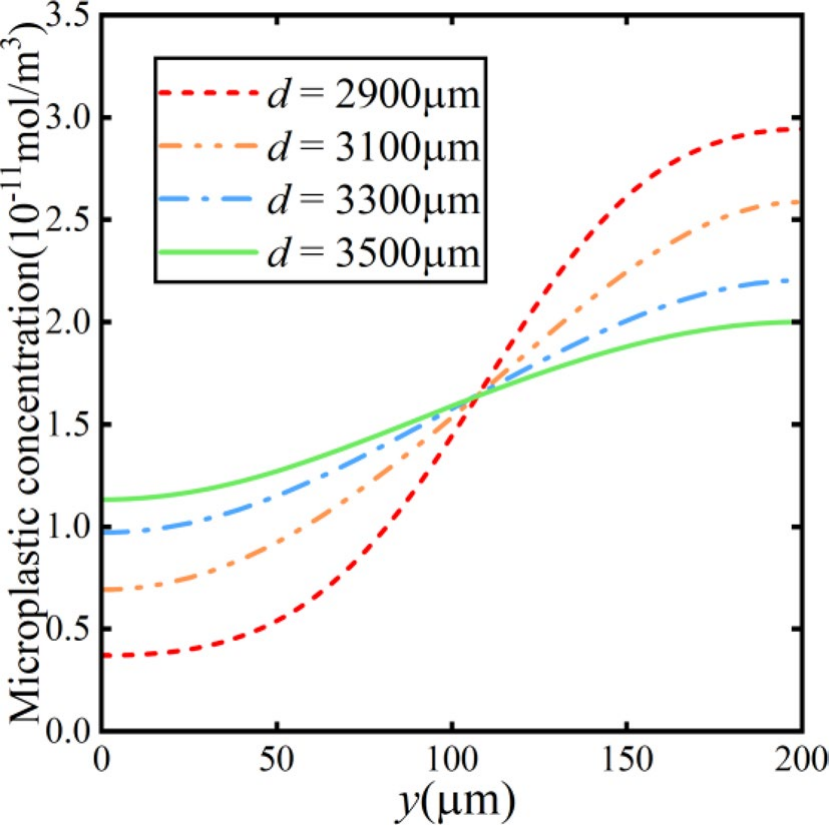
Microplastic concentration along the y-direction at different bipolar electrode distances.

Figure 20 represents the microparticle concentration at *x* = 1900 μm. It is observable that the curve becomes smoother for the increasing distance *d*, indicating a poorer separation efficiency. When the distance* d* is 2900 μm, the ratio of the highest concentration to the lowest concentration can reach 7.95, which shows an effective separation.

**Figure 21:**
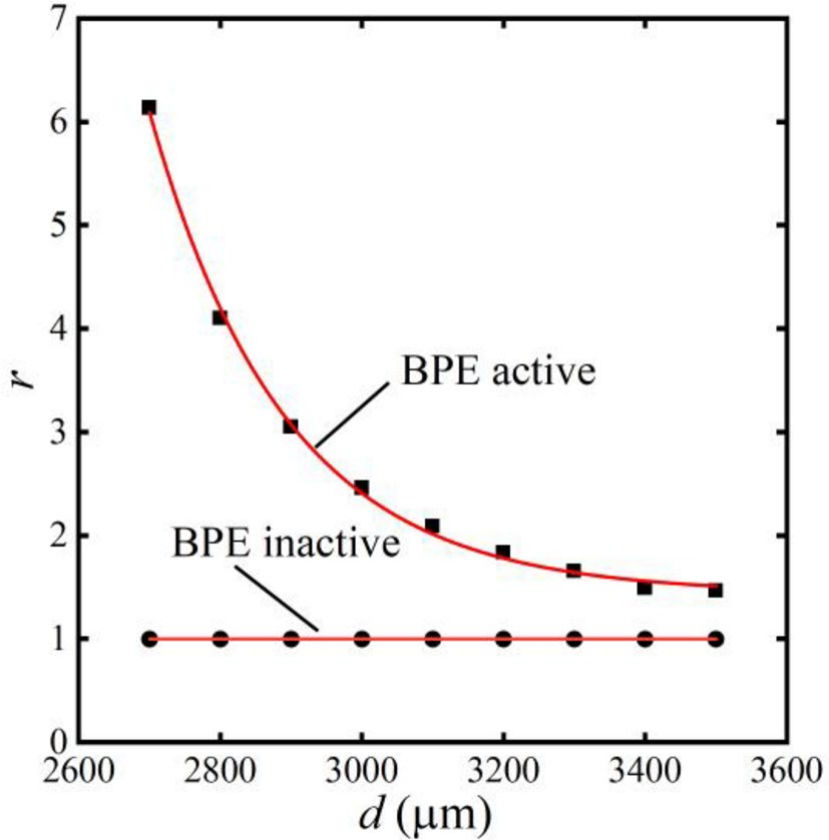
The relationship between separation factor and bipolar electrode distance variation.

Figure 21 illustrates the relationship between the separation factor* r* and the distance *d* between the bipolar electrodes. The expression of this relationship is as follows:

24 $$r \, = \, 6,418,435.891{\text{e}}^{{ - \frac{d}{190.981}}} + 1.44$$

The separation factor exhibits an exponential decay as distance increases in Fig. 21. The electric field is highest near the bifurcation point in the smallest distance, resulting in the strongest electro-migration effect and better separation efficiency. Conversely, the overall increased field strength is unable to achieve effective separation due to being far from the bifurcation point, which leads to a decrease in the separation factor.

To further confirm the separation effect of the microchannels, the separation factor without bipolar electrode action is calculated in Fig. 21. The results show that the separation factor without bipolar electrodes (BPE inactive) is always 1, which means that there is no significant separation effect without bipolar electrodes. On the contrary, the separation factor with the bipolar electrode (BPE active) is higher than 1. This phenomenon indicates that the concentration of the top channel is higher than that of the bottom channel, which means a good effect of separation.

The outlet concentration of microplastics is measured to clearly illustrate the effect of microchannel separation. These results depicted in Table 2 show that the outlet concentration is consistent under the condition of no bipolar electrode. In addition, the top outlet concentration is greater than that of the bottom outlet concentration under the influence of bipolar electrodes. The concentration of the lower channel is reduced to less than half of the original level when the separation factor reaches more than 2, which means an acceptable range in this study.

**Table 2:** The outlet concentration of microplastics (unit: 10^–11^ mol/m^3^).

Concentration	BPE	*d* = 2900 μm	*d* = 3100 μm	*d* = 3300 μm	*d* = 3500 μm
*c*_*m,up*_	Active	2.32	2.06	1.88	1.79
	Inactive	3	3	3	3
*c*_*m,bottom*_	Active	0.76	0.99	1.14	1.22
	Inactive	3	3	3	3

## Conclusions

In this study, we utilized the redox reactions at the bipolar electrode to separate microplastic particles. We employed commercial finite element software to simulate the behavior of microplastic particles in the separation channel. By varying the applied voltage and the separation channel angle, we investigated the influencing factors on microplastic particle behavior. Additionally, we proposed a performance evaluation scheme for separation efficiency.

1. Based on the original separation channel, we propose an improved simplified model of a bifurcated channel utilizing Faradaic electrode reactions under buffer-free solution conditions.
2. Utilizing the finite element method to simulate variations in different parameters, we propose a performance evaluation metric to measure the efficiency of microplastic separation.
3. The simulation results demonstrate that the microchannel can effectively separate microplastic particles under the condition of no buffering solution. Furthermore, with an increase in the applied voltage *V*_*0*_ from 30 to 50 V, the oxidation–reduction reaction rate at the bipolar electrode increases. The concentration of OH^−^ near the cathode increases by approximately 20%, from 4.5 to 5.5 mol/m^3^. Consequently, the separation factor of microplastics also increases by 50%.
4. With an increase in the separation channel angle, there is a slight improvement in the microplastic particle flux in the top channel. However, the separation factor of microplastic particles remains relatively stable with little variation.
5. As the distance *d* between the bipolar electrodes increases, the separation factor *r* exhibits exponential decay. When the distance increases from 2700 to 3500 μm, the separation factor decreases from 6.14 to 1.47, indicating a gradual deterioration in the separation efficiency.

### Supplementary Information

Supplementary Information.

## Data Availability

The datasets used and/or analyzed during the current study are available from the corresponding author on reasonable request. A part of the data generated or analyzed during this study is included in this published article and its supplementary information files.
