# Supplementary material for: Microplastic separation and enrichment in microchannels under derivative electric field gradient by bipolar electrode reactions
Source: Sci Rep. 2024 Feb 26;14:4626. doi: 10.1038/s41598-024-54921-0 (PMC10897390; doi:10.1038/s41598-024-54921-0)
Supplement: Supplementary file 1 — Supplementary Information. [file 41598_2024_54921_MOESM1_ESM.pdf]

Supporting Information

Simulation Parameters

Tables S1-S2 below list the parameter values used in the simulations.

Table S1 The Concentration of Ions in the Solution

Species	concentration	Notes
H^+	10^{-4}[mol/m^3]	the concentration of H^+
OH^-	10^{-4}[mol/m^3]	the concentration of OH^-
K^+	5[mol/m^3]	the concentration of K^+
Cl^-	5[mol/m^3]	the concentration of Cl^-
$Bead^-$	3×10^{-11}[mol/m^3]	the concentration of microplastics
H_2O	5.55×10^{-4}[mol/m^3]	the concentration of H_2O

Table S2 Other Parameters in the Simulation

Parameter	Value	Notes
D_{H}^+	9.103×10^{-9}[m^2/s]	the diffusion coefficient of H^+
D_{OH}^-	5.28×10^{-9}[m^2/s]	the diffusion coefficient of OH^-
D_{K}^+	1.97×10^{-9}[m^2/s]	the diffusion coefficient of K^+
D_{Cl}^-	2.033×10^{-9}[m^2/s]	the diffusion coefficient of Cl^-
D_{Bead-}	7.85×10^{-10}[m^2/s]	the diffusion coefficient of microplastics
Z_{H}^+	1	the number of charges of H^+
Z_{OH}^-	-1	the number of charges of OH^-
Z_{K}^+	1	the number of charges of K^+
Z_{Cl}^-	-1	the number of charges of Cl^-
Z_{Bead}^-	-1	the number of charges of microplastics
ζ	-80[mV]	zeta potential of Electric double layer
ε_r	80	Relative permittivity

Homogeneous Reaction Source Terms

The homogeneous reaction of the microchannel in the manuscript is a water ionization reaction, which can be described by two reaction rate terms. For these forward and reverse reactions, we have the following reaction rates:

$$R_{[OH^-]} = k_f[H_2O] - k_b[OH^-][H^+], \quad (1)$$

$$R_{[H^+]} = k_f[H_2O] - k_b[OH^-][H^+], \quad (2)$$

where k_f and k_b are the forward and reverse reaction rate constants for the ionization reaction of water, respectively. Their values are 10^{-5} s^{-1} and 1.4×10^{11} $m^3/(s\cdot mol)$, respectively. Table S3 shows reaction source terms R_i of species:

Table S3 Reaction Source Terms R_i of Species

Species	Reaction source term R_i
H^+	$R_{[H^+]} = k_f[H_2O] - k_b[OH^-][H^+]$
OH^-	$R_{[OH^-]} = k_f[H_2O] - k_b[OH^-][H^+]$
K^+	0
Cl^-	0
$Bead^-$	0

Numerical simulation

The commercial finite element software Comsol Multiphysics V6.0 was used to solve the equations (3), (5), (6) and (7) in the manuscript. all procedures of the calculation were performed at steady state.

The problem of convention in the microchannel was solved using the “creeping fluid” interface. In fact, the simplified Navier-Stokes equations were calculated in this interface.

The mass transport and the electromigration were calculated by the “Nernst-Planck” interface. We can solve the Nernst-Planck equation under the electroneutrality condition by convection, diffusion, and electromigration.
